# Intrinsic Properties Affecting the Catalytic Activity toward Oxygen Reduction Reaction of Nanostructured Transition Metal Nitrides as Catalysts for Hybrid Na-Air Batteries

**DOI:** 10.3390/ma16237469

**Published:** 2023-12-01

**Authors:** Da Zhang, Kaiwen Zhang, Zhipeng Xie, Bowen Xu, Minjie Hou, Yong Lei, Takayuki Watanabe, Bin Yang, Feng Liang

**Affiliations:** 1 Key Laboratory for Nonferrous Vacuum Metallurgy of Yunnan Province, Kunming University of Science and Technology, Kunming 650093, China; zhangda@kust.edu.cn (D.Z.); 201610113101@stu.kust.edu.cn (K.Z.); 20201102022@stu.kust.edu.cn (Z.X.); 20213102007@stu.kust.edu.cn (B.X.); 20201102007@stu.kust.edu.cn (M.H.); kgyb2005@126.com (B.Y.); 2 National Engineering Research Center of Vacuum Metallurgy, Kunming University of Science and Technology, Kunming 650093, China; 3 Faculty of Metallurgical and Energy Engineering, Kunming University of Science and Technology, Kunming 650093, China; 4 Institute of Physics & IMN MacroNano^®^ (ZIK), Technical University of Ilmenau, 98693 Ilmenau, Germany; yong.lei@tu-ilmenau.de; 5 Department of Chemical Engineering, Kyushu University, Fukuoka 819-0395, Japan; watanabe@chem-eng.kyusu-u.ac.jp

**Keywords:** transition metal nitride, DC arc plasma, ORR, HSABs, intrinsic property

## Abstract

Nanostructured transition metal nitrides (TMNs) have been considered as a promising substitute for precious metal catalysts toward ORR due to their multi-electron orbitals, metallic properties, and low cost. To design TMN catalysts with high catalytic activity toward ORR, the intrinsic features of the influencing factor on the catalytic activity toward ORR of nanostructured TMNs need to be investigated. In this paper, titanium nitride (TiN), zirconium nitride (ZrN), and hafnium nitride (HfN) nanoparticles (NPs) are highly efficient and synthesized in one step by the direct current arc plasma. TiN, ZrN, and HfN NPs with an oxidation layer are applied as the catalysts of hybrid sodium–air batteries (HSABs). The effect of the composition and structural attributes of TMNs on ORR catalysis is defined as follows: (i) composition effect. With the increase in the oxygen content, the catalytic ORR capability of TMNs decreases progressively due to the reduction in oxygen adsorption capacity; (ii) structure effect. The redistribution of the density of states (DOS) of ZrN indicates higher ORR activity than TiN and HfN. HSABs with ZrN exhibit an excellent cyclic stability up to 137 cycles (about 140 h), an outstanding rate performance, and a specific capacity of 2817 mAh·g^−1^ at 1.0 mA·cm^−2^.

## 1. Introduction

Large-scale energy conversion and storage may be addressed by hybrid sodium–air batteries (HSABs), which are advantageous due to their high energy density and plentiful sodium supply [1]. During the operational process of HSABs, one of the active centers of the electrochemical component in HSABs is the oxygen reduction reaction (ORR). However, so far, because O_2_ adhesion on the surface of the electrode, O-O bond activation/cleavage, and oxide elimination are challenging processes, ORR in HSABs has shown slow kinetics, which greatly hinders the usefulness of HSABs [2,3]. Therefore, the successful development of highly efficient electrocatalysts toward ORR will have a dramatic effect on realizing the practical application of HSABs. 

Interestingly, nanostructured transition (TMNs) have become a hot topic because of their high electrical and thermal conductivity, good physical and chemical stability, and exceptional hardness and corrosion resistance [4]. Moreover, they have been considered as a possible replacement for precious metal catalysts toward ORR due to their multi-electron orbitals, metallic characteristics, and low cost, which are attributed to the fact that the redistribution of the density of states (DOS) makes TMNs electron-supplying and produces a catalytic activity similar to that of group VIII noble metals [5,6,7]. Nowadays, researchers mainly focus on the following two aspects of TMNs as catalysts toward ORR. The surface composition of TMN NPs plays a crucial role in the electrocatalytic activity toward ORR due to their improved electrical properties [8,9,10]. Because they facilitate the mass transfer of reactant gasses and ions and promote electrolyte access to the reacting sites, TMN catalysts may provide additional advantages for catalyzing ORR [11,12,13,14]. To design TMN catalysts with high activity toward ORR, it is necessary to simultaneously explore the intrinsic features of these parameters controlling the catalytic activity toward ORR. The right choice of catalyst materials depends on the activity of pure and nanostructured TMNs. However, to improve their effectiveness, a quick and highly effective synthetic approach is also required. TMN nanoparticles (NPs) have been prepared over the last few decades using a variety of methods, including laser ablation [15], arc plasma [16], laser–plasma sputtering [17], direct nitride of transition metals or transition metal oxides [18], ammonia pyrolysis of transition metal chlorides [19], and thermal decomposition of polymer precursors [20]. Among them, arc plasma synthesis has been proven to be a promising route to the efficient production of TMN NPs, because arc plasma can considerably accelerate reaction rates and even motivate reactions that are difficult to execute using conventional techniques.

Based on the research above, the nitrides of the same group of metals were synthesized by the direct current (DC) arc plasma and were considered as the catalysts of HSABs to investigate their ORR capability. Specifically, titanium nitride (TiN), zirconium nitride (ZrN), and hafnium nitride (HfN) NPs were highly efficient and synthesized in one step by a DC arc plasma. The as-synthesized TiN, ZrN, and HfN NPs were applied as HSABs catalysts to evaluate their catalytic activity. Furthermore, the surface composition and defect structure of TMNs played crucial roles in catalyzing ORR by contrasting the structural features of three TMN catalysts.

## 2. Experimental Methods

### 2.1. Preparation of TMN NPs 

The arc plasma equipment was introduced in detail in the published paper [21]. Both the diameter and height of the cylindrically shaped DC arc plasma generator were 300 mm. A tungsten rod (10 mm in diameter) of 99.99% purity (Runde Metal Materials Co., Ltd., Changzhou, China) was used as the cathode, and bulk Ti, Zr, and Hf of 99.99% purity (Runde Metal Materials Co., Ltd., Changzhou, China) were used as anodes. Given that the anode is continuously consumed during the discharge process, the tungsten rod was positioned perpendicular to the anodic center and was able to travel toward it in order to maintain two electrodes 5–10 mm apart. Prior to the injection of 50 kPa nitrogen with a purity of 99.99% (Messe, Frankfurt, Germany) into the arc plasma apparatus, the chamber pressure was reduced to less than 3 Pa. Initial nitrogen as a buffer gas with 60 kPa pressure was employed to synthesize the TMN NPs, and the discharge current was maintained at 200 A. The inner-wall sediment was collected after discharging. 

### 2.2. The Cyclic Voltammogram (CV) and Linear Sweep Voltammetry (LSV) Performance Test

A three-electrode system was used to measure rotating disk electrode (RDE) voltammetry (MSR, PINE, Pittsburgh, PA, USA), with Ag/AgCl and platinum flack serving as the reference and counter electrodes, respectively, and the reversible hydrogen electrode (RHE) receiving all potentials. A quantity of 5 mg of TMNs was ultrasonically mixed with 500 mL ethyl alcohol, 450 mL deionized water, and 50 mL of 5 weight percent Nafion solution to create the catalyst ink, which was then left to sit for 30 min. A glassy carbon disk with a diameter of 5.0 mm was polished, and 20 mL of suspension was dropped onto it. The disk was then allowed to dry at an ambient temperature. Using a 0.1 M potassium hydroxide (KOH) solution, all electrochemical experiments were carried out in RDE electrochemical cells. Positive-going potential sweeps from −0.9 to 0.3 V were used to carry out CV at 10 mV·s^–1^. Positive-going potential sweeps from −0.8 to 0.2 V at 10 mV·s^–1^ were used to investigate ORR activity.

### 2.3. Alkali Leaching Experiment

To test the ability of TiN, ZrN, and HfN NPs to resist alkali corrosion, 100 mg of each sample was placed into 100 mL 0.5 M sodium hydroxide (NaOH) solution and kept at room temperature for 24 h. Thereafter, the morphology and structure of the three kinds of solutions were directly tested.

### 2.4. Preparation of Air Electrode 

In HSABs, air electrodes (the cathode) consist of carbon papers and catalysts. Carbon papers were pretreated to promote electrical conductivity: 10 mg carbon black (EBORY, Guangzhou, China) was added into 10 mL polytetrafluoroethylene (PTFE, 5–10 wt%) and then treated with ultrasound for 40–60 min. Pristine carbon papers (Toray, Tokyo, Japan) were soaked in a homogeneous suspension for 20 min. After drying out at room temperature, the treated carbon papers were insulated in the high-temperature fox furnace at 450 °C for 1 h. A quantity of 15 mg TMN NPs mixed with 4 mg carbon black were poured into a 2 mL conical test tube and next injected with ethanol and PTFE. The suspension was dripped onto the surface of 7 pieces of pretreated carbon paper. Carbon papers decorated with TMN NPs served as air electrodes to test the properties of the HSABs.

### 2.5. Assembly of HSABs 

A 0.5 M NaOH solution was employed as an aqueous catholyte, while 1 M NaClO_4_ in EC/DMC (1:1) with 1 vol% FEC (Mojiesi Energy Company, Beijing, China) was considered as a nonaqueous anolyte. These two solutions were prevented from intermixing via a separating membrane of a solid-state electrolyte Na_3_Zr_2_Si_2_PO_12_ (NASICON). The synthesis process of NASICON was introduced in detail in our previous study [22]. HSABs were built using the following components: metallic sodium, anolyte, NASICON, catholyte, catalytic cathode [23].

### 2.6. Electrochemical Performance Test 

A LAND battery tester (CT2001A; Wuhan LAND electronics, Wuhan, China) was used to perform discharge/charge testing on HSABs at 30 °C. CV and LSV were achieved by using a CHI 630 electrochemical analyzer combined with an RDE system (Princeton Applied Research; Model 616). 

### 2.7. Characterization

A transmission electron microscope (TEM, JEM-2100; JEOL, Tokyo, Japan), high-resolution TEM (HRTEM), and selected area electron diffraction (SAED) with a 200 kV accelerating voltage were used to examine the morphology of the TMNs. Using Cu-Kα radiation and X-ray diffraction (XRD, Rigaku D/Max-2500; Rigaku, Tokyo, Japan) at 5 º·min^–1^, the crystalline structure was characterized in the 2θ range of 20 to 80°. The chemical bonding state was examined using an X-ray photoelectron spectroscopy (XPS; Kratos Axis Ultra DLD, Hadano, Japan) equipped with an Al Kα line (*hv* = 1486.6 eV). A surface area analyzer (V-Sorb X800; Gold APP Instruments, Beijing, China) was used to measure the N_2_ absorption/desorption isotherms of TMNs. The Brunauer–Emmett–Teller (BET) method was employed to determine the samples’ specific surface area (SSA).

## 3. Results and Discussion

**Figure 1:**
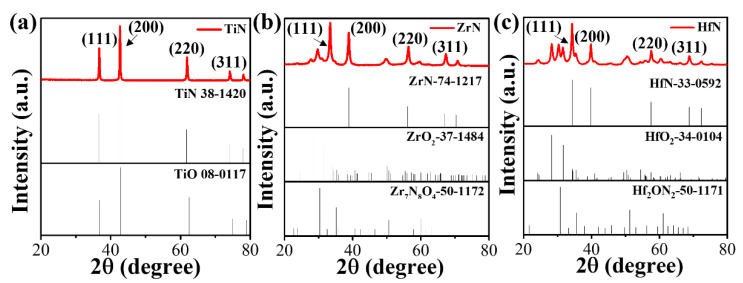
XRD patterns of the samples synthesized by a DC arc plasma with (**a**) Ti, (**b**) Zr, and (**c**) Hf bulk as anodes.

The XRD patterns of TiN, ZrN, and HfN synthesized by a DC arc plasma are shown in Figure 1. The sample consists of a cubic TiO phase (JCPDS card No. TiO 08-0117) and cubic TiN phase (JCPDS card No. TiN 38-1420), as can be seen from Figure 1a, and these XRD peaks are attributed to (111), (200), (220), and (311) of TiN [24]. In particular, as shown in Figure S1, the peak belonging to TiO is the shoulder peak near the distinctive peak of TiN. As seen in Figure 1b, the sample contains a cubic ZrN phase (JCPDS Card No. ZrN 74-1217), the monoclinic ZrO_2_ phase (JCPDS card no. ZrO_2_ 37-1484), and the Zr_7_O_8_N_4_ phase (JCPDS Card No. Zr_7_O_8_N_4_ 38-1420). The appearance of the ZrO_2_ phase and the Zr_7_O_8_N_4_ phase is attributed to the reaction between ZrN and air, and these XRD peaks are attributed to (111), (200), (220), and (311) of ZrN [25]. Remarkably, the sample comprises a cubic HfN phase (JCPDS card No. HfN 33-0592), cubic HfO_2_ phase (JCPDS card No. HfO_2_ 34-0104), and Hf_2_ON_2_ phase (JCPDS card No. Hf_2_ON_2_ 50-1171) in Figure 1c. HfO_2_ and Hf_2_ON_2_ are derived from the reaction between HfN and air. Similar to TiN and ZrN, these XRD peaks are attributed to (111), (200), (220), and (311) of HfN [26]. Ultimately, the formation of oxygen and zirconium vacancies in the monoclinic ZrO_2_ phase leads to significant decreases in the lattice parameter of ZrN, which possesses higher ORR activity than the cubic TiO and HfO_2_ phases [27]. 

**Figure 2:**
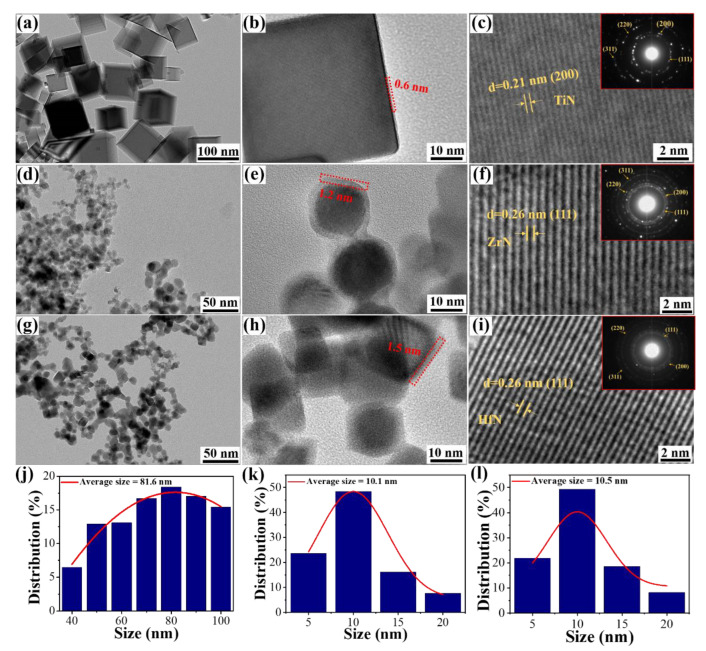
The TiN’s (**a**) TEM image, (**b**,**c**) HRTEM images, with inset SAED image; the ZrN’s (**d**) TEM image, (**e**,**f**) HRTEM images, with inset SAED image; the HfN’s (**g**) TEM image, (**h**,**i**) HRTEM images, with inset SAED image. The images of (**j**) TiN, (**k**) ZrN, and (**l**) the HfN size distributions.

Figure 2a shows TEM images of TiN, demonstrating that all TiN NPs show a regular cubic structure, good dispersion, and uniform particle size. Based on the HRTEM image in Figure 2b, the reaction between TiN and air produces an initial oxide layer on the surface of the as-synthesized TiN NPs that has a thickness of 0.6 nm. Figure 2c shows the interplanar distance of cubic TiN NPs, indicating that the as-synthesized TiN has good crystallinity and a 0.21 nm lattice spacing, consistent with the cubic TiN (200) crystal plane [28]. The SAED pattern implies that the as-obtained TiN is highly crystalline, and these XRD peaks are attributed to (111), (200), (220), and (311) of TiN [29]. Figure 2d exhibits a TEM image of ZrN, suggesting that ZrN NPs have a near-spherical morphology with few intergrowths and good dispersion. As inferred from the HRTEM image of ZrN NPs in Figure 2e, ZrN NPs with a thickness of 1.2 nm are generated by the reaction between ZrN and air. It is found that ZrN NPs have good crystallinity and a 0.26 nm lattice spacing, corresponding to the ZrN crystal plane (111) [30]. According to the SAED analysis of ZrN, these XRD peaks are attributed to (111), (200), (220), and (311), as shown in the inset of Figure 2f [31]. Figure 2g shows a TEM image of HfN, in which HfN NPs have a near-spherical morphology with few intergrowths and good dispersion. The HRTEM image of HfN in Figure 2h displays an oxidation layer with a thickness of 1.5 nm from the reaction between HfN and air. Moreover, HfN NPs have excellent crystallinity, and their lattice spacing is 0.26 nm, corresponding to the (111) crystal plane [32]. According to the SAED of HfN, these XRD peaks are attributed to (111), (200), (220), and (311), as shown in the inset of Figure 2i [33]. TEM analysis of over 200 particles is used to study the size distribution of TiN, ZrN, and HfN NPs. The TiN’s size range in Figure 2j is 40–100 nm, and the average size is 81.6 nm. It can be seen from the size distribution in Figure 2k that the size range of the as-synthesized ZrN NPs is 5–20 nm, and the average size is 10.1 nm. The size range of HfN NPs is 5–20 nm, with an average size of 10.5 nm, as illustrated in Figure 2l. TiN NPs have larger crystal sizes than ZrN and HfN NPs, suggesting that TiN electrodes may suffer from the less exposed active sites toward ORR [34].

**Figure 3:**
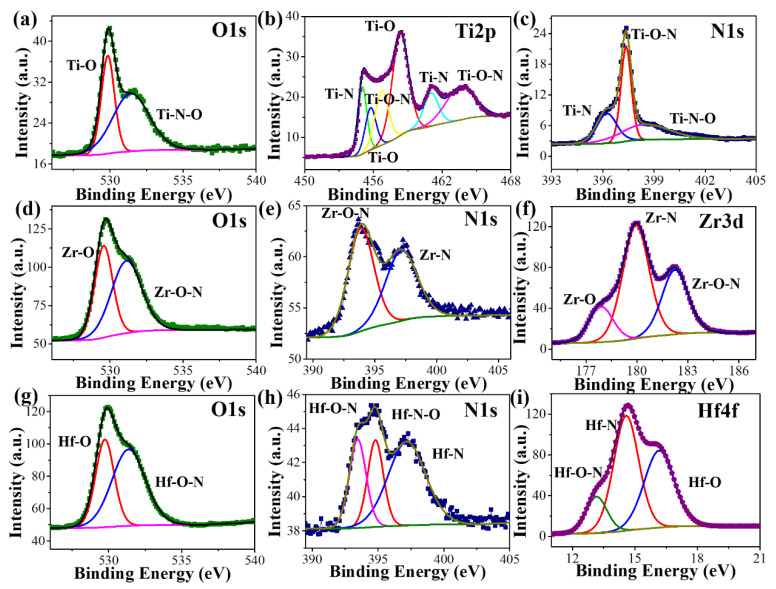
(**a**) O1s, (**b**) Ti 2p, and (**c**) N1s XPS spectra of TiN; (**d**) O1s, (**e**) N1s, and (**f**) Zr 3d XPS spectra of ZrN; and (**g**) O1s, (**h**) N1s, and (**i**) Hf 4f XPS spectra of HfN.

The deconvolution of Ti 2p XPS is used to analyze the surface composition of TiN, as illustrated in Figure 3b. This is demonstrated by the Ti 2p XPS spectrum at 455.0 eV and 461.1 eV for Ti-N, 456.8 eV and 458.4 eV for Ti-O, and 455.8 eV and 463.8 eV for Ti-O-N, respectively. The O 1s (Figure 3a) and N 1s (Figure 3c) XPS spectra of TiN provide additional support for this conclusion. The O 1s peaks at 530.1 eV and 532.2 eV are attributed to Ti-O and Ti-N-O, respectively, as displayed in Figure 3a. As can be seen in Figure 3c, Ti-N is responsible for the N 1s peak at 395.8 eV, while Ti-O-N and Ti-N-O are responsible for the other two peaks at 397.8 eV and 398.5 eV, respectively [35,36,37]. The surface composition in Figure 3f of ZrN is analyzed using the deconvolution of the Zr 3d XPS spectrum. Peak fitting is evidenced by the Zr 3d XPS spectra at 177.8 eV related to Zr-O-N, at 179.9 eV related to Zr-N, and at 182.2 eV related to Zr-O, respectively. This is further affirmed by the N 1s and O 1s XPS spectra of ZrN. Detailed information about the O 1s and N 1s core levels is discussed in Figure 3d,e, in which Zr-N-O is tested at 393.2 eV and 532.2 eV, and Zr-N is confirmed by the peak at 397.4 eV, while Zr-O is evidenced by the peak at 532.2 eV [38,39,40]. The peaks at 13.2 eV, 14.6 eV, and 16.2 eV in Figure 3i could be attributed to Hf bonded with N or O, and the existing formations of Hf are Hf-O, Hf-N, and Hf-O-N under the Hf 4f XPS spectrum, which is further evidenced by the N 1s and O 1s XPS spectra of HfN. The 529.7 eV and 532.1 eV peaks are assigned to the O 1s peak (Figure 3g) of the Hf-O and Hf-O-N species, respectively. The N1s peak (Figure 3h) can be decomposed into three components: 393.3 eV for Hf-O-N, 394.8 eV for Hf-N-O, and 397.2 eV for Hf-N, respectively [41,42,43,44]. According to the TEM results, oxygen-containing functional groups of three samples arise from the oxidation layer at the edge of TMNs. The XRD, XPS, and TEM results suggest that TiN, ZrN, and HfN NPs with an oxidized structure can be deemed to have a structural defect, which has definite catalytic activity toward ORR [45]. Furthermore, the degree of oxidation correlates with the oxygen’s interaction with the catalyst surface, while the catalytic activity toward ORR decreases when the oxygen content is above 8% [46,47]. As illustrated in Figure S2, the oxygen content of the HfN sample is far higher than that of the other two TMNs, suggesting that the HfN NPs catalyst has poorer ORR performance. The oxide thickness of three TMNs confirms the oxygen content, i.e., 1.5 nm for HfN, 1.2 nm for ZrN, and 0.6 nm for TiN. 

BET analysis is achieved by detecting the SSA of TiN, ZrN, and HfN NPs through their N_2_ adsorption/desorption isotherms. As shown in Figure S3, all isotherms show close to IV-type patterns based on IUPAC classification [48]. The SSA of TiN, ZrN, and HfN NPs is 34.2 m^2^·g^−1^, 69.6 m^2^·g^−1^, and 62.3 m^2^·g^−1^, respectively. The phenomenon is put down to the particle size of TiN, ZrN, and HfN NPs, which is confirmed by their size distribution in Figure 3. Additionally, the IV-type isotherm at 0.2–1.0 *P*/*Po* exhibits a steep rise in adsorption and a minor hysteresis between the adsorption/desorption branches, indicating the presence of numerous mesopores and macropores with uniform porosity distribution [49,50]. 

**Figure 4:**
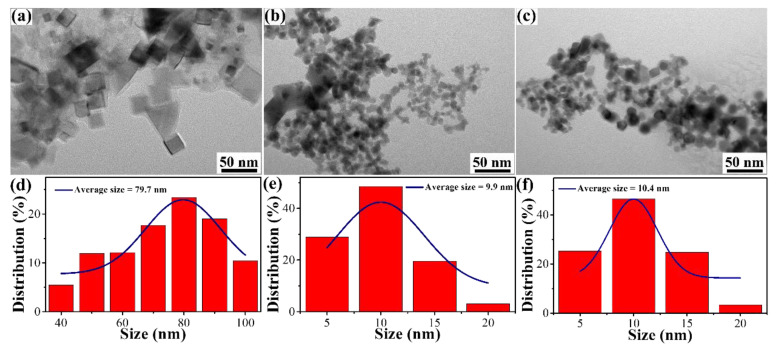
(**a**–**c**) TEM images and (**d**–**f**) size distribution images of TiN, ZrN, and HfN treated with 0.5 M NaOH solution.

TMNs easily react with a hydroxyl ion (i.e., MN + 3OH^−^→ NH_3_ + ${MO}_{3}^{3-}$, M = Ti, Zr, and Hf), suggesting that TMNs cannot be used as a catalyst of HSABs with an alkaline solution electrolyte. However, TiN, ZrN, and HfN NPs synthesized by a DC arc plasma are coated with an oxidation layer. To investigate the stability of the resistance to alkaline solutions, as shown in Figure 4, TiN, ZrN, and HfN NPs are corroded in 0.5 M NaOH solution for 24 h. It can be obviously noted that the morphology and structure of TiN, ZrN, and HfN NPs have not changed. To further illustrate morphologies, the size distribution of TiN, ZrN, and HfN NPs was studied. It can be seen from the size distribution of TiN, ZrN, and HfN in Figure 4d–f that the average size of the as-synthesized TiN, ZrN, and HfN NPs is 79.7 nm, 9.9 nm, and 10.4 nm, respectively. The unaltered structure and morphology signify that TiN, ZrN, and HfN NPs are able to resist corrosion in the alkaline solution due to the presence of the oxidation layer. Therefore, the three TMNs as mentioned earlier can be used as stable catalysts for HSABs with an alkaline solution electrolyte.

**Figure 5:**
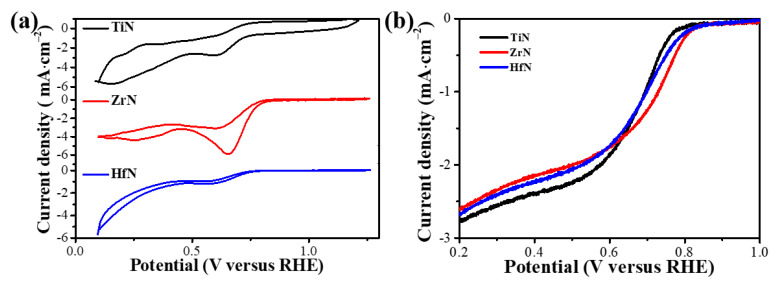
(**a**) CV curves of TiN, ZrN, and HfN NP catalysts in an O_2_-saturated 0.1 M KOH solution; (**b**) ORR polarization curves of TiN, ZrN, and HfN NP catalysts at 1600 rmp at a scan rate of 10 mV s^−1^.

To examine ORR performance, the CV curves of TiN, ZrN, and HfN NP catalysts in an O_2_-saturated 0.1 M KOH solution were measured with the same amount of the active material. These ORR peaks can be found easily in an O_2_-saturated solution at a voltage between 0 and 1.0 V, indicating a pronounced electrocatalytic activity in TiN, ZrN, and HfN catalysts toward ORR. As shown in Figure 5a, the peaks at 0.64, 0.71, and 0.67 V vs. the RHE for TiN, ZrN, and HfN NP catalysts correspond to the reversible redox reaction. It can be seen that ZrN NPs have better catalytic performance than TiN and HfN NPs catalysts. To further explore the oxygen reduction ability of the three substances, ORR polarization curves of TiN, ZrN, and HfN as catalysts were measured in 0.1 M KOH solution by LSV at various rotation velocities, as shown in Figure S4a–c. As illustrated in Figure 5b and Table S1, the onset potential and half-wave potential of TiN, ZrN, and HfN catalysts are 0.78 and 0.62 V, 0.82 and 0.7 V, and 0.8 and 0.67 V, respectively, at a rotating speed of 1600 rpm. By fitting the K–L point diagram of JL-1 to ω^–1/2^ under different voltages, the number of electrons transferred is computed by following the straight line’s slope. As shown in Figure S4d–f, the transferred electron number range of the TiN, ZrN, and HfN catalysts is 1.62–2.01, 2.67–2.78, and 2.35–2.74, respectively, indicating that the main product of ORR is H_2_O_2_ [51]. Based on the previous report, all lattices are perfectly symmetrical in cubic microstructure, leading to the same oxygen adsorption capacity [7]. However, given the synergistic effect between surface oxide species and Zr sites, and a higher SSA than TiN and HfN, ZrN outperforms other TMN catalysts in terms of the electrochemical properties of HSABs.

**Figure 6:**
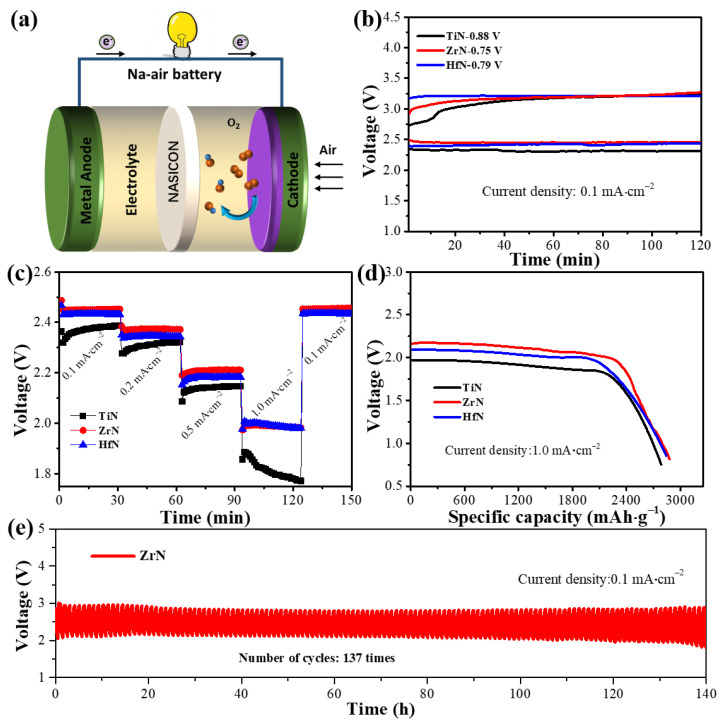
(**a**) Schematic illustration of HSABs with TiN, ZrN, and HfN NPs as catalysts, electrochemical performances of the proposed HSABs; (**b**) discharge and charge curves with different catalysts at 0.1 mA·cm^−2^; (**c**) profiles of charge and discharge at various current densities; (**d**) discharge capacities curves at 1.0 mA·cm^−2^; and (**e**) the cycling performance of HSABs with ZrN NPs as catalysts at 0.1 mA·cm^−2^.

HSABs are assembled using carbon paper with loaded TMN NP catalysts to investigate the electrochemical performance. A typical structure of TiN, ZrN, and HfN NPs used as catalysts in HSABs is depicted in Figure 6a. Metallic Na loses electrons during the discharge process to become Na^+^ (4Na – 4e → 4Na^+^), which travels to the cathode via the anolyte and NASICON. Meanwhile, Na^+^ combines with the electron and OH^−^ that is obtained from the ORR to form sodium hydroxide at the cathode (O_2_ + 2H_2_O + 4e → 4OH^−^). During the charge process, metallic Na is plated at the anode and O_2_ is evolved at the cathode [1]. The electrochemical properties of HSABs with TiN, ZrN, and HfN catalysts using 0.5 M NaOH as electrolytes was investigated. Figure 6b exhibits the discharge and charge curves of TiN, ZrN, and HfN as catalysts of HSABs at 0.1 mA·cm^–2^. The voltage gap of 0.75 V ZrN and 0.79 V HfN catalysts is smaller than that of 0.88V TiN catalysts, which is mainly reflected in the catalytic performance toward ORR. The discharge and charge profiles of HSABs with TiN, ZrN, and HfN catalysts at various current densities are displayed in Figure 6c. At current densities of 0.1, 0.2, 0.5, and 1.0 mA·cm^−2^, the discharge end voltages of HSABs with a ZrN catalyst are 2.46, 2.38, 2.18, and 1.86 V, respectively. In addition, the discharge terminal voltage can be restored to 2.48 V once the density of current returns to 0.1 mA·cm^−2^, showing an excellent rate performance, which may be due to the ideal electrochemical environment and catalytic activity of ZrN NPs. As shown in Figure 6d, ZrN NP catalyst-equipped HSABs have a higher specific capacity of 2817 mAh·g^−1^ at 1.0 mA·cm^−2^ than TiN (2659 mAh·g^−1^) and HfN (2785 mAh·g^−1^). Figure 6e shows the discharge and charge profiles of ZrN NPs. The stability of HSABs with ZrN NP catalysts is examined by performing constant current discharge and charge with up to 137 cycles (about 140 h). The validation of the cathodic potential for real-world uses of rechargeable batteries is largely dependent on this procedure. In the case of continuous discharge and charge of the battery, no significant potential drop was observed. 

**Figure 7:**
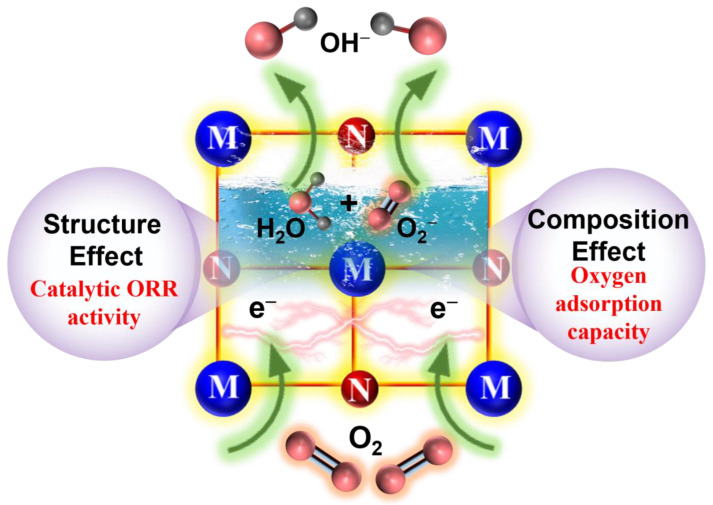
Intrinsic properties affecting the catalytic activity toward ORR of TiN, ZrN, and HfN NPs as catalysts of HSABs.

Figure 7 shows the intrinsic properties affecting the catalytic activity toward ORR of TiN, ZrN, and HfN NPs as catalysts of HSABs. Nanostructured TMNs could provide an extra advantage for the electrolyte to enter the reaction site, improving the O_2_ and ion-conducting mass transfer in the catalyst layer [5]. Combined with the preceding analysis, the mechanism of TMN catalysts toward ORR could be explained as follows: (i) Composition effect. This contributed to the synergistic effect between surface oxide species and metal sites; TMNs demonstrate excellent ORR performance because incorporated oxygen compensates for the charge imbalance caused by nitrogen atoms [48]. However, the maximal ORR activity is achieved through the minimization of the bulk oxygen content in cubic TMNs due to a strong driving force for the oxygen adsorption, signifying that the ORR catalytic performance of TMNs decreases gradually with increasing oxygen content when the oxygen content is above 8% [47]. The ORR catalytic performances of TMNs decrease progressively with increasing oxygen content due to diminishing of the oxygen adsorption capacity. ZrN, with much lower oxygen content than HfN, suggests that a ZrN catalyst exhibits a better ORR performance; (ii) Structure effect. The monoclinic ZrO_2_ phase with oxygen and zirconium vacancies results in a significant decrease in the lattice parameter of ZrN, signifying that the redistribution of DOS enhances ORR catalysis ability. In this case, the defect form of TMNs plays a crucial role in ORR catalytic performance.

## 4. Conclusions

In conclusion, TiN, ZrN, and HfN NPs are highly efficient and synthesized in one step by a DC arc plasma. Specifically, the average size and SSA of TiN NPs, with regular cubic structure, are 81.6 nm and 34.2 m^2^·g^−1^, respectively. The average size and SSA of ZrN NPs with a near-spherical structure are 10.1 nm and 69.6 m^2^·g^−1^, respectively. The average size and SSA of HfN NPs with a near-spherical morphology are 10.5 nm and 62.3 m^2^·g^−1^, respectively. Due to the presence of the oxidation layer, TiN, ZrN, and HfN NPs can resist corrosion in an alkaline solution, suggesting that these TMNs can be used as a catalyst for HSABs with an alkaline solution electrolyte. Indeed, HSABs with a ZrN catalyst exhibit a voltage gap of 0.75 V, excellent rate performance, and a specific capacity of 2817 mAh·g^−1^ at 1.0 mA·cm^−2^, which is superior to HSABs with TiN and HfN catalysts. The affecting factors of TMNs catalysis toward ORR, such as lower oxygen content leading to strong oxygen adsorption capacity and the redistribution of DOS from defects boosting ORR catalytic ability, are finally validated. This work expands the utilization of a DC arc plasma in synthesizing TMN NPs, and may also provide feasible strategies to help design advanced TMN electrocatalysts toward ORR.

## Data Availability

The data that support the findings of this study are available on request from the corresponding author upon reasonable request.

## Supplementary Materials

The following supporting information can be downloaded at: https://www.mdpi.com/article/10.3390/ma16237469/s1, Figure S1: XRD pattern of TiN synthesized by DC arc discharge; Figure S2: XPS spectra of the as-synthesized TiN, ZrN, and HfN NPs; Figure S3: N_2_ adsorption/desorption isotherms curves of TiN, ZrN, and HfN NPs; Figure S4: In 0.1 M KOH solution at different rotating rates with a current density of 10 mV·s^−1^, (a) ORR po-larization curve of TiN and (d) K-L point plot calculating the number of transferred electrons, (b) ORR polarization curve of ZrN and (e) The number of transferred electrons, and (c) ORR polarization curve of ZrN and (f) The number of transferred electrons; Table S1: Onset potentials and half-wave potentials of TiN, ZrN, and HfN as catalysts toward ORR.
