# Supplementary material for: Intrinsic Properties Affecting the Catalytic Activity toward Oxygen Reduction Reaction of Nanostructured Transition Metal Nitrides as Catalysts for Hybrid Na-Air Batteries
Source: Materials (Basel). 2023 Dec 1;16(23):7469. doi: 10.3390/ma16237469 (PMC10707049; doi:10.3390/ma16237469)
Supplement: Supplementary file 1 [file materials-16-07469-s001.zip › materials-2580379-supplementary.pdf]

Intrinsic Properties Affecting the Catalytic Activity toward Oxygen Reduction Reaction of Nanostructured Transition Metal Nitrides as Catalysts for Hybrid Na-Air Batteries

Da Zhang [1,2,3,†], Kaiwen Zhang [1,2,3,†], Zhipeng Xie [1,2,3], Bowen Xu [1,2,3], Minjie Hou [1,2,3], Yong Lei [4], Takayuki Watanabe [5], Bin Yang [1,2,3] and Feng Liang [1,2,3,*]

1 Key Laboratory for Nonferrous Vacuum Metallurgy of Yunnan Province, Kunming University of Science and Technology, Kunming 650093, China; zhangda@kust.edu.cn (D.Z.); 201610113101@stu.kust.edu.cn (K.Z.); 20201102022@stu.kust.edu.cn (Z.X.); 20213102007@stu.kust.edu.cn (B.X.); 20201102007@stu.kust.edu.cn (M.H.); kgyb2005@126.com (B.Y.)

2 National Engineering Research Center of Vacuum Metallurgy, Kunming University of Science and Technology, Kunming 650093, China

3 Faculty of Metallurgical and Energy Engineering, Kunming University of Science and Technology, Kunming 650093, China

4 Institute of Physics & IMN MacroNano® (ZIK), Technical University of Ilmenau, 98693 Ilmenau, Germany; yong.lei@tu-ilmenau.de

5 Department of Chemical Engineering, Kyushu University, Fukuoka 819-0395, Japan; watanabe@chem-eng.kyusu-u.ac.jp

* Correspondence: liangfeng@kust.edu.cn; Tel./Fax: +86-871-6510720

† These authors contributed equally to this work.

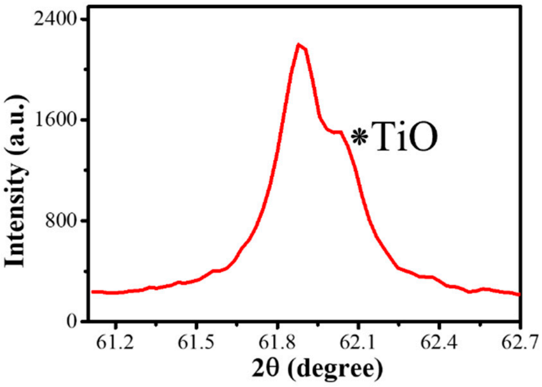

Figure S1. XRD pattern of TiN synthesized by DC arc discharge.

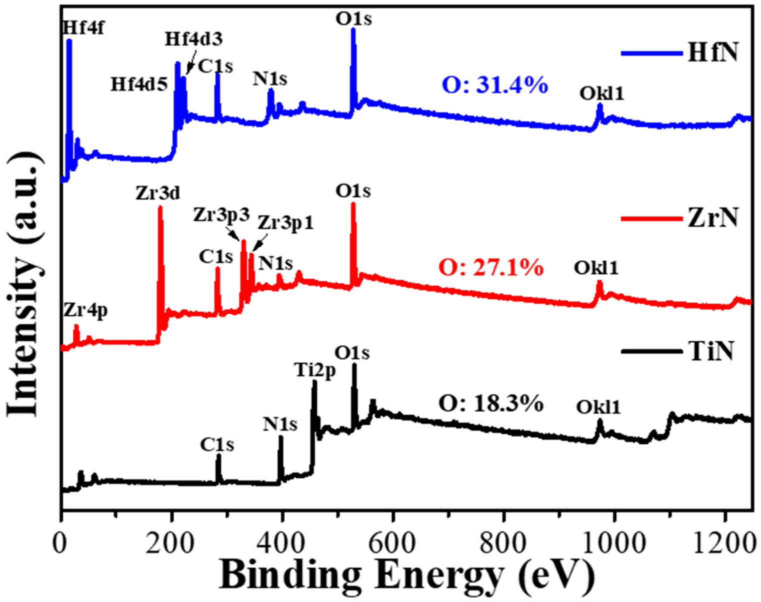

Figure S2. XPS spectra of the as-synthesized TiN, ZrN, and HfN NPs.

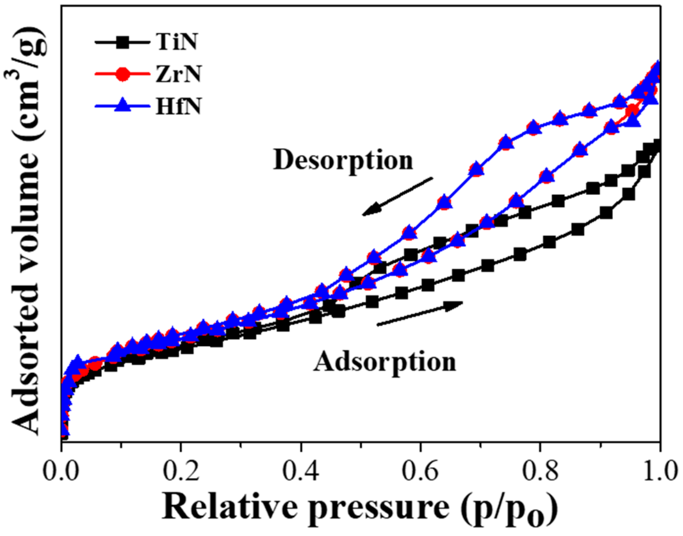

Figure S3. N_2 adsorption/desorption isotherms curves of TiN, ZrN, and HfN NPs.

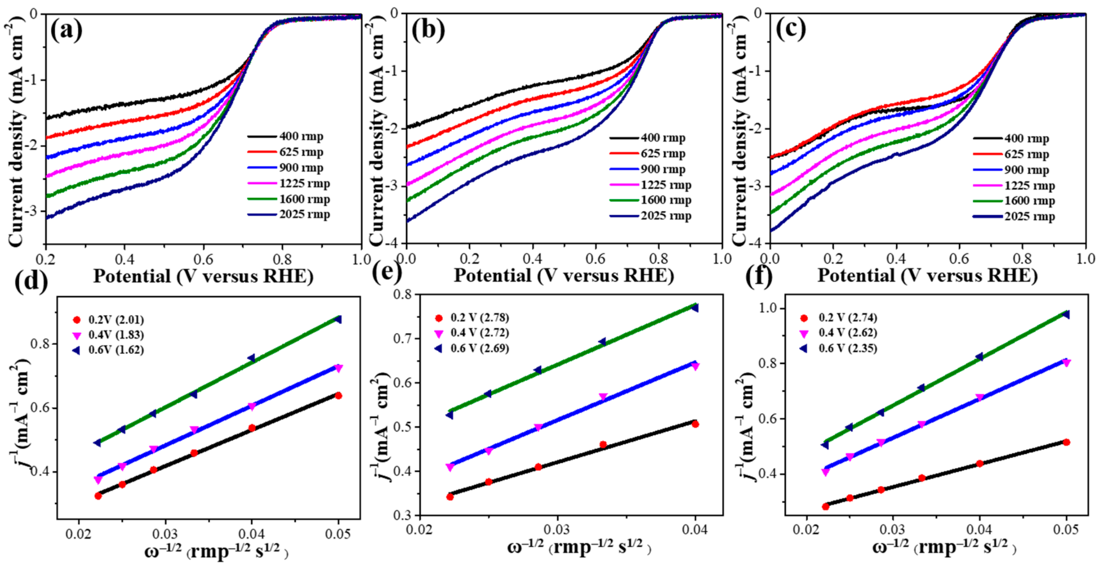

Figure S4. In 0.1 M KOH solution at different rotating rates with a current density of 10 mV·s^{-1}, (a) ORR polarization curve of TiN and (d) K-L point plot calculating the number of transferred electrons, (b) ORR polarization curve of ZrN and (e) The number of transferred electrons, and (c) ORR polarization curve of ZrN and (f) The number of transferred electrons.

Table S1. Onset potentials and half-wave potentials of TiN, ZrN, and HfN as catalysts toward ORR.

Catalyst	Onset potentials (V)	Half-wave potentials (V)
TiN	0.78	0.65
ZrN	0.82	0.7
HfN	0.8	0.67
